# One for all, all for one - Establishing a corporate linkage methodology for integrated health analytics in Canada

**DOI:** 10.23889/ijpds.v1i1.340

**Published:** 2017-04-19

**Authors:** David Paton, Lori Kirby, Philippe Poitras

**Affiliations:** 1 Canadian Institute for Health Information

## Objectives

To describe the successes, challenges and trade-offs encountered when establishing a standard patient linkage methodology for linking pan-Canadian health data across the continuum of care.

## Approach

Health facilities, regional health authorities and ministries of health across Canada need access to timely, integrated health information across the continuum of care in order to make effective decisions to manage and improve their health systems. To better meet these needs, our organization has embarked on an initiative to integrate our web-based analytic tools. A requirement for this integration is the establishment of a corporate linkage standard.

The scope is to enable linkage across a dozen patient-level data holdings including data from acute care, long term care, home care, rehabilitation, mental health, pharmaceuticals and registries for joint replacements and organ transplants. In Canada, provinces and territories issue jurisdiction-specific health care numbers (HCN) to their residents.

A working group was established to review existing methodologies and to define a standard linkage. To gain support from the various groups in our organization to adopt the proposed standard, the right balance was sought between accuracy, timeliness, ease of implementation as well as availability and quality of data elements across our data holdings. In addition, an accurate patient linkage key was manually created as a benchmark to compare false positives and false negatives of the various candidate methods.

## Results

In general, adding data elements to the linkage methodology increased false negatives, decreased false positives and reduced the number of records that could be included in the linkage. Data quality issues affecting linkages varied across the data holdings and by jurisdiction.

The final standard linkage methodology is simple and deterministic: link records by jurisdiction and HCN and exclude records where HCN is used by multiple people, for example, newborns who share their HCN with their mother.

## Conclusion

Arriving at a methodology that balanced the need for inclusiveness, simplicity and precision required collaboration, compromise, analysis and innovation. To date, the new client linkage standard has been implemented for ad-hoc analysis as well as in the data warehouse for our new web analytics tool. The implementation of the new client linkage standard represents an important foundational step towards creating an integrated analytics tool that spans across the continuum of care.

